# Minimally invasive accessory splenectomy for recurrent gastric variceal bleeding due to left-sided portal hypertension: report of the first case

**DOI:** 10.1093/jscr/rjab008

**Published:** 2021-02-11

**Authors:** S C Schmidt, J Möller, N Bürgel, C Radke, L Beyer, F Marusch

**Affiliations:** Department for Hepato-Pancreato-Biliary Surgery, Ernst von Bergmann Clinic, Potsdam, Germany; Department for Hepato-Pancreato-Biliary Surgery, Ernst von Bergmann Clinic, Potsdam, Germany; Department for Gastroenterology and Infectiology, Ernst von Bergmann Clinic, Potsdam, Germany; Institute for Pathology, Ernst von Bergmann Clinic, Potsdam, Germany; Department for Radiology, Ernst von Bergmann Clinic, Potsdam, Germany; Clinic for General-, Visceral-, Vascular- and Thoracic Surgery, Ernst von Bergmann Clinic, Potsdam, Germany

## Abstract

Upper gastrointestinal bleeding from esophagogastric varices is a common scenario, especially in patients with portal hypertension induced by liver cirrhosis or other diseases with thrombosis of the splenic vein. However, accessory spleen as pathophysiological cause of a regional, left-sided portal hypertension and consecutive development of isolated gastric varices is rare. We report a case of recurrent gastric variceal bleeding resulting from sinistral portal hypertension associated with an accessory spleen in a patient who had traumatic splenectomy many decades before. The accessory spleen is an extremely rare cause for the development of regional, left-sided portal hypertension leading to isolated gastric varices. Minimally invasive splenectomy is a safe and efficient treatment option.

## INTRODUCTION

Accessory spleens are not so uncommon with a reported incidence between 3% and 18% [1]. Mostly they are asymptomatic. However, in patients with relapse of idiopathic thrombocytopenia after initial splenectomy, evaluation of an accessory spleen should be undertaken [2]. Bleeding from gastric varices due to accessory spleen-related localized sinistral portal hypertension is extremely rare and to our knowledge only described in a patient with concomitant liver cirrhosis so far [3]. In the present paper, we report on a patient who suffered refractory gastric variceal bleeding 60 years after posttraumatic splenectomy caused by a giant accessory spleen.

**Figure 1:**
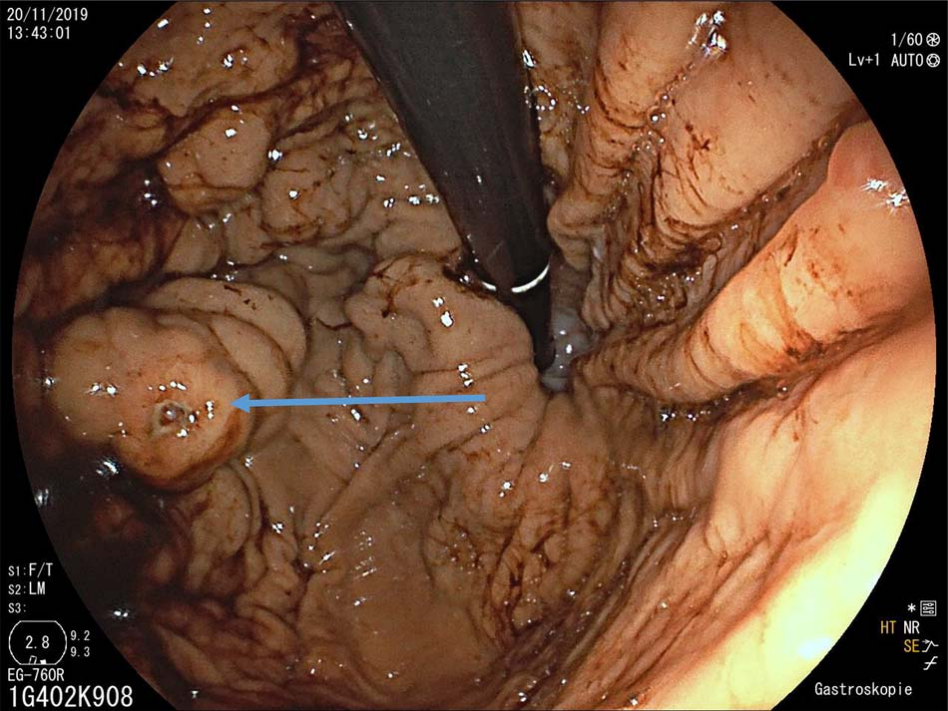
Upper gastrointestinal endoscopy: gastric varices (arrow) in the fundus after previous bleeding.

## CASE REPORT

A 65-year-old man with recurrent upper gastrointestinal bleeding was admitted to the Department of Internal Medicine in our institution to identify the source of the varices and therapy. The patient had two episodes of acute upper gastrointestinal bleeding within 1 year, which was treated by sclerosing of gastric varices in a primary hospital. An important note in his medical history was a splenectomy 6 decades before after splenic rupture by blunt abdominal trauma. Endoscopy of the upper gastrointestinal tract showed isolated gastric fundus varices with the absence of oesophageal varices (Fig. 1). To identify the source of the gastric varices, an enhanced computed tomography (CT) of the abdomen was performed and surprisingly demonstrated an 8 cm in diameter mass, mimicking an accessory spleen which

**Figure 2:**
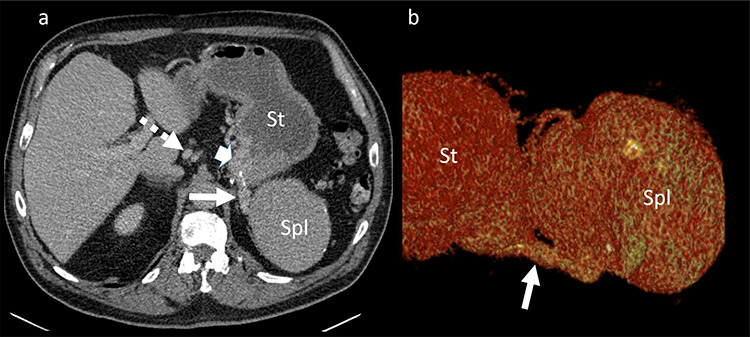
Portal venous phase CT of the upper abdomen (**a**) with a 3D reconstruction (**b**); note the gastric varices (arrowhead) and dilatation of short gastric veins (solid arrow) and coronary vein (dashed arrow); St: stomach; Spl: accessory spleen.

was located in the left upper quadrant of the abdomen, nearly adherent to the stomach and a moderate variceal conglomeration in the fundus of the stomach (Fig. 2). Liver cirrhosis or portal vein occlusion and other inflammatory or malignant diseases could be excluded from CT imaging. In addition, a CT scan was able to show clearly that the arterial blood supply of the accessory spleen is derived from the short gastric arteries. Splenic artery and vein could not be delineated. The patient was subsequently presented to our Department of Visceral Surgery. We decided to perform laparoscopic accessory splenectomy to relief the regional hypertension of the short gastric veins. In the operation room, the patient was positioned in right semi-decubitus position. One 12 mm Trocar, two 11 mm Trocars and on 5 mm Trocar were inserted in the left upper quadrant. The accessory spleen was extremely adherent to the diaphragm and retroperitoneal tissue (Fig. 3). After transection of the short gastric vessels with the vessel sealer (Ligasure™, Medtronic, Germany), the spleen was mobilized and removed (Fig. 4). Patient’s postoperative course was completely uneventful, and he was discharged in good health condition on the seventh postoperative day. The diagnosis was confirmed by immunohistologic examination (Fig. 5).

**Figure 3:**
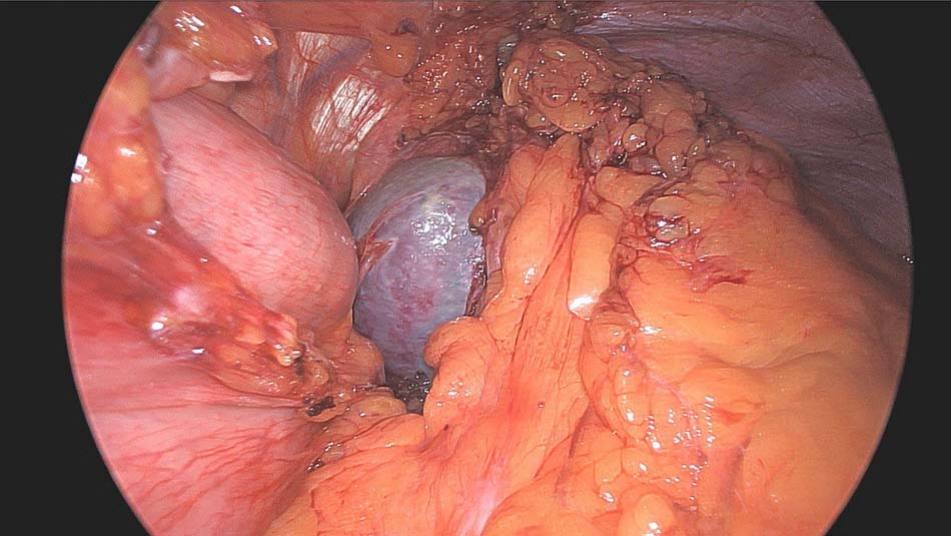
Intraoperative finding of the accessory spleen.

**Figure 4:**
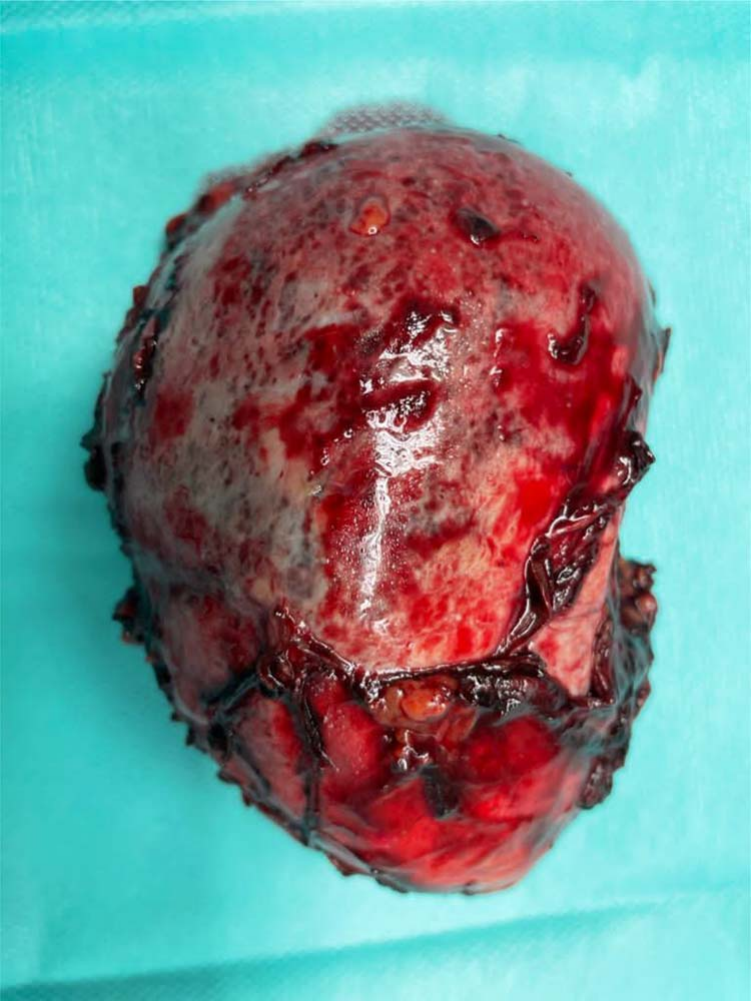
Resected specimen.

**Figure 5:**
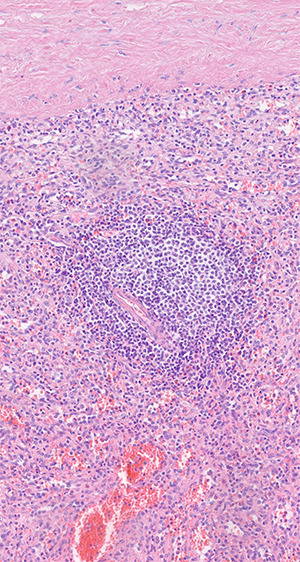
Histologic examination shows red and white pulp as main compartments of the spleen; B-cell follicle with geminal center, mantle- and marginal-zone; perifollicular red pulp with sinus; HE×20.

## DISCUSSION

**Figure 6:**
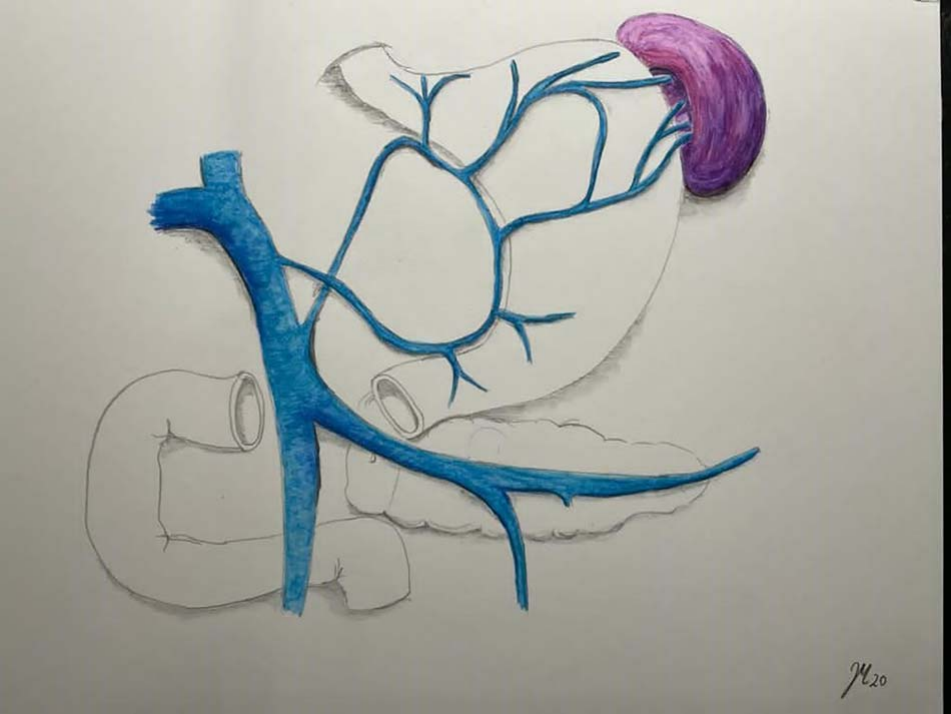
Pathophysiology of left side portal hypertension with gastric varices.

The development of isolated gastric varices is rare and associated with regional, left-sided or sinistral portal hypertension [4]. Left-sided portal hypertension mostly results from pancreatitis induced splenic vein thrombosis [5]. Other, but rare etiological factors of localized sinistral hypertension include regional inflammation, malignancies of the hepato-pancreato-biliary system but also injuries of the splenic vein following liver transplantation, distal gastrectomy or minimally invasive pancreatic tail resection [6]. In the reported patient, left-sided portal hypertension was the result of an accessory spleen that drained the venous blood through the short gastric veins to the fundus. The arterial blood flow to the accessory spleen probably arised from the gastro-omental artery and short gastric arteries. Venous blood reflux through the short gastric veins led to an increase of the intravenous pressure with subsequent development of gastric varices at the fundus (Fig. 6). Regional hypertension led to an enormous enlargement of the accessory spleen. Accessory spleen develops either from spilled splenic tissue at the initial operation or hypertrophy of small splenic rest tissue. As a congenital defect, the accessory spleen was also described to be missed at the initial splenectomy [7]. Accessory spleens are usually small solid masses < 4 cm in diameter. Although compensatory hypertrophy after removal of the principal spleen is not uncommon, the finding of such a large accessory spleen as in our case is rather atypical.

The diagnostic management of patients with isolated gastric varices in the fundus may be a challenge. Endoscopy is an experienced center is necessary since gastric varices at the fundus may easily be overlooked because they may be hidden by rugal folds [4]. To identify the underlying pathology of the gastric varices and—like in our case—to visualize the blood supply of the accessory spleen as well as the venous conglomerate between the accessory spleen and the fundus a contrast-enhanced abdominal computed tomography scan is indispensable.

Treatment of patients with bleeding gastric varices from left-sided portal hypertension includes endoscopic sclerotherapy in combination with either endovascular embolization or surgery of the underlying disease [8]. Accessory splenectomy for gastric variceal bleeding has been only described in one case report until now and this patient also had liver cirrhosis [3]. Transarterial embolization is an option [9]. We performed splenectomy since we estimated the risk for subsequent partial gastric infarction as high.

## CONCLUSION

The accessory spleen as the cause of left-sided regional portal hypertension leading to recurrent bleeding of isolated fundic varices seems to be a very rare constellation. The patient presented in this case report was successfully managed by minimally invasive accessory splenectomy. To our knowledge, this is the first case with bleeding gastric varices on the basis of left sides portal hypertension due to accessory spleen reported in the literature until now.
